# Construction of dominant rice population under dry cultivation by seeding rate and nitrogen rate interaction

**DOI:** 10.1038/s41598-021-86707-z

**Published:** 2021-03-30

**Authors:** Hao Jiang, Tebogo Thobakgale, Yunzhe Li, Liwei Liu, Qingwang Su, Baifeng Cang, Chenyang Bai, Jiayi Li, Ze Song, Meikang Wu, Dongchao Wang, Jingjing Cui, Xiaoshuang Wei, Zhihai Wu

**Affiliations:** 1 Agronomy College, Jilin Agricultural University, Changchun, 130118 Jilin China; 2 College of Life Sciences, Jilin Agricultural University, Changchun, 130118 Jilin China; 3 Changchun JINONGDA Agricultural Technology Company Limited, Changchun, 130118 Jilin China

**Keywords:** Plant sciences, Photosynthesis, Plant physiology

## Abstract

This study used the rice cultivar Suijing 18 to investigate the effects of morphological characteristics, photosynthetic changes, yield, as well as nitrogen absorption and utilization. The interaction between seeding rate and nitrogen rate was also assessed to identify the most suitable values of the dominant population for both factors under dry cultivation. Furthermore, the photosynthetic physiological characteristics of the upper three leaves in the dominant population were also explored. The results showed that a combination of 195 kg/ha seeding rate and 140 kg/ha nitrogen rate achieved high yield, high nitrogen utilization, and moderate morphological characteristics. This was achieved by a coordination of the combined advantages of population panicle number and spikelets per panicle. The photosynthetic potential of the population was improved by coordinating the reasonable distribution of light energy in the upper three leaves, which led to the emergence of a dominant rice population under dry cultivation.

## Introduction

Increasing temperatures and water scarcity as a result of climate change have become urgent global challenges. Growing population, increasing agricultural water demand, and decreasing availability of freshwater resources have exacerbated the impact of drought on agricultural production, thus making the study of sustainable agricultural production models critical^1^. Rice is at the core of food crops^2^, and the exploration of simplified cultivation technologies for rice has received much attention from scholars both in China and internationally^3–5^. Dry cultivation of rice is a rice crop mode which differs from transplanted rice, water direct seeding, wet direct seeding, and dry direct seeding water pipe methods. Dry cultivation represents a rice crop mode that does not include seedling and transplanting, but rather, rice is directly sown under dry land preparation conditions. This mode mainly relies on natural precipitation during the whole reproductive period, and water is only appropriately replenished during critical periods of high water demand or in times of drought. Dry cultivation of rice has been rapidly developing in different rice-cultivating regions both in China and internationally. This method eliminates the need for seedling breeding and transplanting, saves water resources, improves labor efficiency, and optimizes both land use efficiency and adaptation to mechanization. Both seeding rate and nitrogen rate are important cultivation measures that affect rice yield formation and population composition^6,7^. It has been shown that increasing nitrogen rate and density can increase the effective panicle number and plant number in rice populations, while the increase of panicle number and spikelets per panicle are mutually constraining^8^. Under the condition of reciprocal seeding rate and nitrogen rate, increasing the seeding rate may offset the negative effect of reducing the nitrogen rate on yield. Both nitrogen rate and seeding rate exert a significant effect on the interception of leaf area index and photosynthetically active radiation, which are two important factors in the formation of seed yield^9^. Numerous studies have shown several ways to improve N recovery efficiency (NRE) in rice. A 20–39% reduction in nitrogen rate has been shown to reduce nitrogen losses by 21–45% and ensure 95–99% of the maximum possible yield^10,11^. Selection of varieties with high nitrogen utilization is an effective means to reduce nitrogen losses^12^. A suitable canopy structure is an important characteristic of high yielding and highly efficient rice cultivars. The reasonable configuration of leaf length and leaf angle of the upper three leaves is closely related to yield^13,14^. Numerous previous studies have assessed the effects of seeding and nitrogen variation on dryland crop populations of e.g., cotton^15^, oilseed rape^16^, maize^17^, and wheat^18^. The light and simplified rice cultivation, mostly from the perspective of variety, sowing period, density, residues^19^, fertilizers^20^, and mechanized planting methods^21^, fully shows that the measures of each production link significantly impact the construction of the dominant population of light simplified cultivation^22–24^. This also highlights the importance of canopy photosynthetic capacity^25,26^. Previous studies mostly measured and optimized the characteristics of crop population from the perspective of a single factor. At present, many scholars have constructed dominant crop populations from the perspective of two factors and multi factor interactions^27,28^. The influence of photosynthetic coordination of different leaf positions on crop population has also attracted strong attention. However, the construction of dominant rice populations under dry cultivation based on two-factor interactions have not been investigated, and neither have the photosynthetic characteristics of different leaf positions under the interaction of seeding rate and nitrogen rate. To fill this gap, this study used the rice cultivar Suijing 18 to study the effects of different leaf position morphological characteristics, photosynthetic changes, yield, as well as nitrogen absorption and utilization. The interaction between seeding rate and nitrogen rate was assessed to identify the most suitable seeding rate and nitrogen rate allocation of the dominant population under dry cultivation. Furthermore, the photosynthetic physiological characteristics of the upper three leaves of the dominant population were explored under dry cultivation. The results provide population optimization methods and breeding objectives for high-yielding and high-efficiency light-simplified rice cultivation patterns under dry cultivation. Moreover, this paper provides both a theoretical basis and technical support for the construction of high-light-efficient populations.

## Results

### Yield and yield components

The effects of seeding rate, nitrogen rate, and the interaction effect of both on yield reached significant levels (*P* = 0.001; *P* < 0.01; *P* = 0.011). The mean yield under each nitrogen rate was highest in the A4 population. The highest yielding combinations at each seeding rate were A1B5, A2B5, A3B4, A4B5, and A5B5 (see Table 1), and the plants at seeding rates A4 and A5 had severely collapsed at the filling stage after the nitrogen rate had reached B4. The A4B3 combination achieved high yield, within A2B5 > A3B4 > A4B3 > A1B5 > A5B3.

**Table 1:** Effects of interaction between seeding rate and nitrogen rate on yield and yield components of rice under dry cultivation.

N rate (kg·ha^−1^)	Seeding rate (kg·ha^−1^)	Panicles per m^2^	Spikelets per panicle	Filled grains (%)	1000-grain weight (g)	Grain yield (kg·ha^−1^)
A1	B1	284.7 ± 10.1 a	69.5 ± 1.2 a	94.4 ± 1.1 a	25.2 ± 0.6 a	4700.8 ± 86.7 b
	B2	318.3 ± 17 a	83.1 ± 2.3 a	92.9 ± 1.7 a	26 ± 0.3 a	6395.7 ± 139 ab
	B3	370.3 ± 15.9 a	80.7 ± 1.2 a	93.1 ± 0.7 a	25 ± 0.5 a	6960.3 ± 174.2 ab
	B4	321.7 ± 5.6 a	76.5 ± 2.8 a	93.6 ± 0.5 a	26.2 ± 0.8 a	6049.9 ± 159.6 ab
	B5	390 ± 13.9 a	83.3 ± 1.2 a	95.4 ± 1.2 a	25.7 ± 1.8 a	7967.2 ± 231.7 a
	Mean	337	78.6	93.9	25.6	6414.8
A2	B1	221.7 ± 6.8 c	69 ± 2 a	91.6 ± 2 a	24.6 ± 0.6 a	3452.2 ± 159.2 c
	B2	417 ± 10.2 ab	77.1 ± 2.6 a	94.9 ± 1.1 a	24.9 ± 0.7 a	7587.7 ± 152.8 b
	B3	381.3 ± 18.9 b	80.9 ± 3.2 a	93.4 ± 1.7 a	26.1 ± 1.4 a	7512.3 ± 228.6 b
	B4	411.7 ± 17 ab	72.8 ± 1.9 a	95.8 ± 1 a	25.1 ± 2 a	7202.5 ± 85 b
	B5	511.7 ± 20.5 a	84.5 ± 1.1 a	93.5 ± 1.3 a	24.4 ± 1 a	9843.4 ± 202.9 a
	Mean	388.7	76.9	93.8	25	7119.6
A3	B1	267.3 ± 20.5 b	53.8 ± 3.1 c	94.9 ± 1.6 a	24.8 ± 0.6 a	3379.3 ± 167.1 c
	B2	285 ± 11.2 ab	68.7 ± 2.1 b	88.5 ± 1.5 a	26 ± 1.3 a	4497.5 ± 105 bc
	B3	407 ± 9.5 ab	75.5 ± 0.9 b	91.4 ± 0.2 a	24.4 ± 0.6 a	6846.6 ± 79.5 ab
	B4	447.7 ± 16.5 a	91.2 ± 3.8 a	90.4 ± 1.1 a	24 ± 0.6 a	8866.5 ± 181.2 a
	B5	417.3 ± 18.7 ab	79.5 ± 2.5 ab	93.4 ± 0.5 a	25.2 ± 1 a	7805.5 ± 208.4 a
	Mean	364.9	73.7	91.7	24.9	6279.1
A4	B1	340.7 ± 20.6 a	59.9 ± 3.4 d	90.6 ± 1.1 a	23.4 ± 0.7 b	4331.5 ± 189.4 c
	B2	504 ± 16.3 a	71.9 ± 2.4 bc	94 ± 2.4 a	25.2 ± 0.4 ab	8581.7 ± 157.3 ab
	B3	445.3 ± 21.8 a	81.6 ± 1.4 ab	93.2 ± 1.7 a	25.9 ± 1.1 a	8763.9 ± 207.3 ab
	B4	471.3 ± 24.5 a	67.9 ± 1.9 cd	93.5 ± 2.8 a	24.8 ± 1.6 ab	7426.4 ± 181 b
	B5	498 ± 11.5 a	87.3 ± 1.3 a	95.8 ± 1 a	25.2 ± 0.3 ab	10,499.8 ± 230.4 a
	Mean	451.9	73.7	93.4	24.9	7920.7
A5	B1	497.7 ± 17.2 b	40 ± 1.4 b	91 ± 1.1 b	24.7 ± 1.6 a	4469.1 ± 216.8 a
	B2	421.7 ± 25.2 b	66.9 ± 3.3 a	94.6 ± 1.6 ab	25.2 ± 1.3 a	6710.7 ± 138 ab
	B3	512.3 ± 5.6 b	62.1 ± 2.5 a	95.9 ± 1.5 a	26 ± 0.2 a	7950.1 ± 221.2 a
	B4	491.7 ± 23.3 b	72.4 ± 3.3 a	91.5 ± 2.6 b	24.8 ± 1 a	8065 ± 207.4 a
	B5	619 ± 11.8 a	60.6 ± 2.1 a	91.5 ± 1.2 b	24.5 ± 1.3 a	8422.9 ± 129 a
	Mean	508.5	60.4	92.9	25	7123.6
Variance analysis	A	**(F = 19.346)	**(F = 22.643)	*(F = 3.391)	ns(F = 1.030)	**(F = 5.744)
	B	**(F = 14.654)	**(F = 29.413)	ns(F = 1.087)	ns(F = 2.217)	**(F = 42.873)
	A × B	*(F = 2.196)	**(F = 4.388)	**(F = 3.473)	ns(F = 1.267)	*(F = 2.365)

A: Seeding rate, B: N rate. A1: 60 kg·ha^−1^, A2: 105 kg·ha^−1^, A3: 150 kg·ha^−1^, A4: 195 kg·ha^−1^, and A5: 240 kg·ha^−1^; B1: 0 kg·ha^−1^, B2: 70 kg·ha^−1^, B3: 140 kg·ha^−1^, B4: 210 kg·ha^−1^, and B5: 280 kg·ha^−1^. The values represent the mean ± SD of three replicates. Different letters with the same sowing amount indicate a significant difference at P < 0.05. * and ** indicate that the yield components are significantly influenced by the N rate, seeding rate, and their interactions at 0.05 and 0.01 levels, respectively, and ns indicates “not significant”. This applies to all following figures.

Increasing the seeding rate increased the average population panicle number, while it decreased the average number of spikelets per panicle. The degree of response to nitrogen changes varied at different seeding rates; increasing the nitrogen rate at A1 and A2 seeding rates increased the panicle number and spikelets per panicle and achieved high yield. A3 seeding rate under B4 nitrogen rate achieved the highest panicle number and spikelets per panicle and obtained high yield. The number of spikelets per panicle was lower under A4 and A5 seeding rates, and the increased nitrogen rate resulted in higher yields mainly by increasing the population panicle number.

### External features

**Figure 1:**
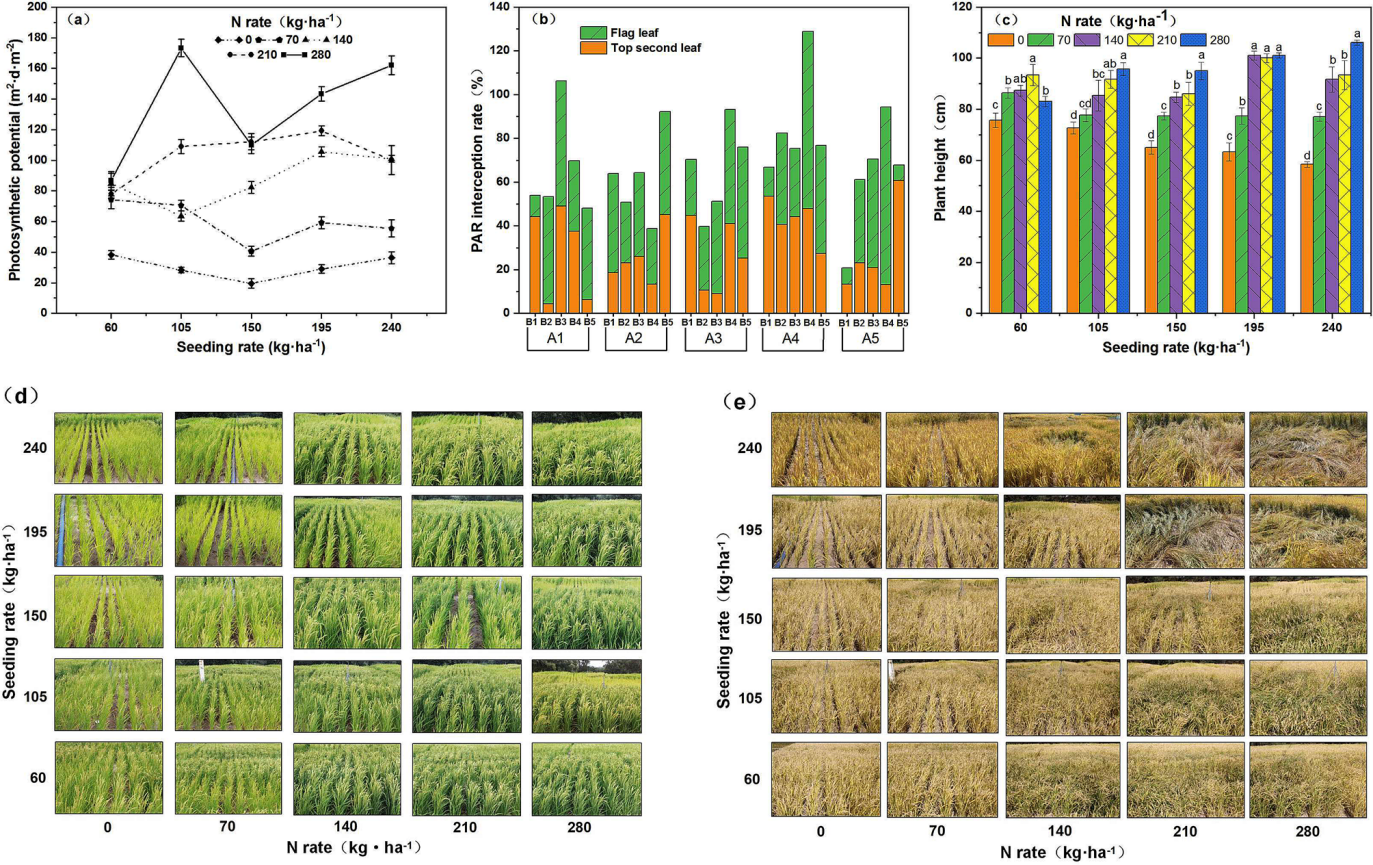
Effects of the interaction between seeding rate and nitrogen rate on the photosynthetic potential of rice, PAR interception rate at different leaf positions, and plant height 10 days after flowering under dry cultivation. (**a**) Photosynthetic potential; (**b**) interception rate of light energy at different leaf positions; (**c**) plant height; (**d**) pictures of plant height using Origin software to process and merge pictures. (**e**) Rice growth after flowering under dry cultivation. (**f**) Growth of rice at the mature stage under dry cultivation. Different letters with the same sowing amount indicate a significant difference at P < 0.05. The figures were prepared and combined using Origin 2018 (www.originlab.com).

The mean levels of photosynthetic potential, plant height, and light energy interception were highest among seeding rates in the A4 population. The photosynthetic potential increased with increasing nitrogen rate and was higher at B4 and B5 at all seeding rates, which was consistent with the pattern identified for yield trends. The average light energy interception rate of the population followed an inverted second leaf > flag leaf trend, with the highest values occurring in the range of B3–B5 at all seeding rates. Plant height followed an overall increasing trend with increasing nitrogen rate, compared with the increase at B1, which gradually increased with increasing seeding rate (Fig. 1). The area of population lodging reached 80% and the nitrogen rate reached up to B4 at A4 and A5 seeding rates. The photosynthetic potential reached 119.19 m^2^ day/m^2^ at A4 seeding rate, and the plant height reached 101 cm with associated risk of lodging. The photosynthetic potential reached 100.05 m^2^ day/m^2^ at A5 seeding rate and the plant height reached 93.33 cm with associated risk of lodging. The coordination of photosynthetic potential, light energy interception capacity of different leaf positions, and plant height of the A4B3 population under interaction conditions was reasonable. A photosynthetic potential of 105.55 m^2^·day/m^2^, a 31.21% light energy interception in the inverted second leaf, and a plant height of 101 cm 10 days after flowering were important external characteristic of the dominant population under dry cultivation.

### Net photosynthetic rate and chlorophyll content

**Figure 2:**
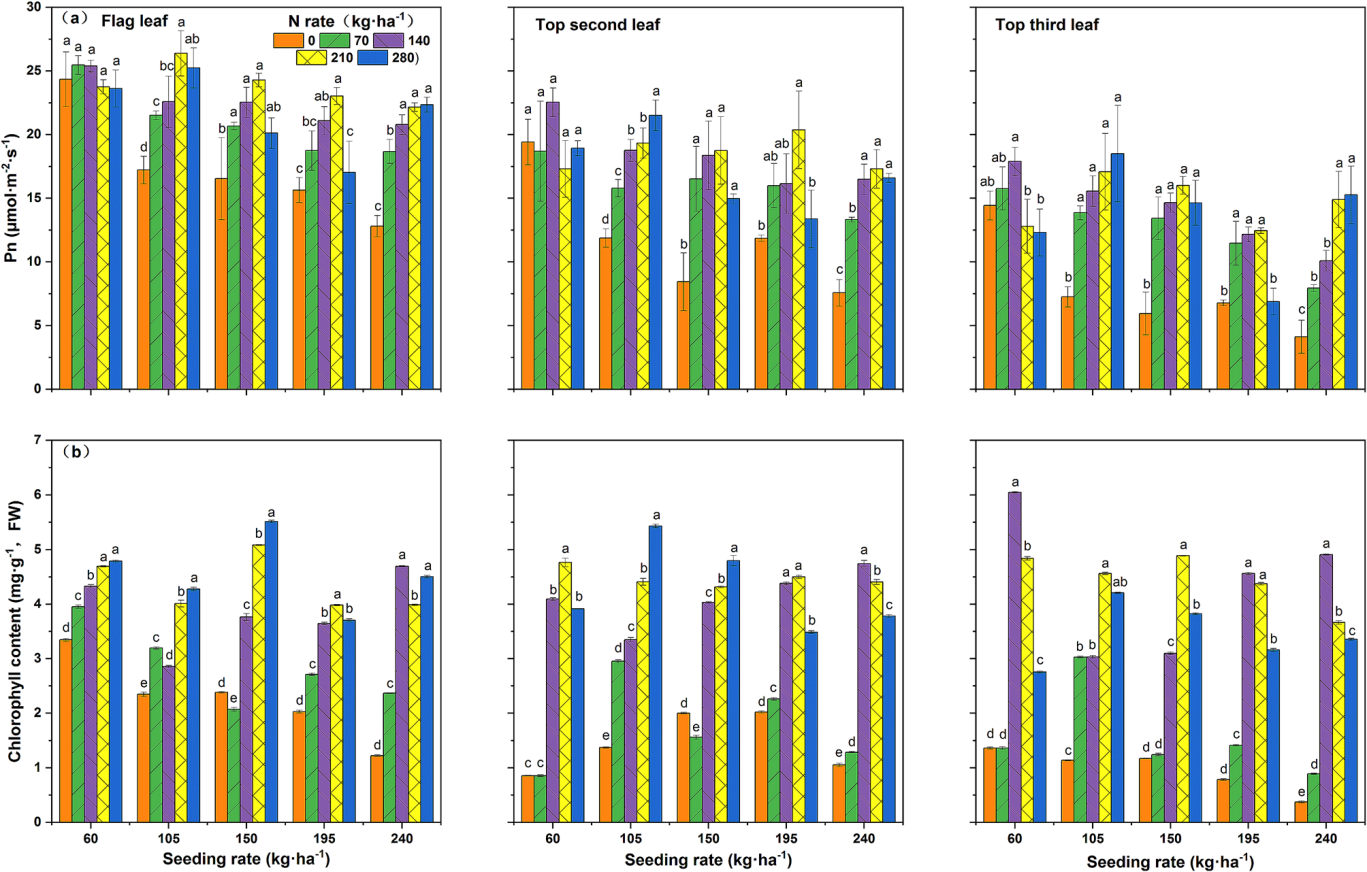
Effects of the interaction between seeding rate and nitrogen rate on the net photosynthetic rate and chlorophyll content for different leaf positions in the rice population 10 days after flowering under dry cultivation. (**a**) Net photosynthetic rate of the flag leaf; first second leaf and the first third leaf. (**b**) Chlorophyll content of the flag leaf; first second leaf and first third leaf. Different letters with the same sowing amount indicate significant differences at P < 0.05. The figures were prepared and combined using Origin 2018 (www.originlab.com).

With increasing seeding rate, both Pn(net photosynthetic rate) and Chl(chlorophyll) in the upper three leaves followed a decreasing trend Pn and Chl at different leaf positions under each seeding rate were higher in the flag leaf and the inverted second leaf. These results showed that the Pn and Chl levels of the population could be improved by increasing the nitrogen rate; however, the response degree of different leaf positions to nitrogen change differed in response to different seeding rates. When the seeding rate reached A4–A5, continued increase in nitrogen rate after reaching B3 would cause decreases or a leveling off in the flag leaf Pn and Chl. The Pn and Chl trends in the inverted second leaf and the inverted third leaf were identical. Under A3–A5 seeding rates, Pn reached a higher level at B4. Chl was higher at B3–B4 levels under A4–A5 seeding rates, in response to the continuing increase of the nitrogen rate, Pn and Chl followed a stable or decreasing trend. In terms of the interaction between nitrogen rate and seeding rate, the highest values of Pn and Chl in all three leaf positions were in the range of B3–B5. Under A4 seeding rate, the total Pn and Chl of the A4B4 combination were the highest, and A4B3 also reached a higher level (Fig. 2).

### Rubisco(ribulose-1,5-bisphosphate carboxylase) and RCA(Rubisco activase) activity

**Figure 3:**
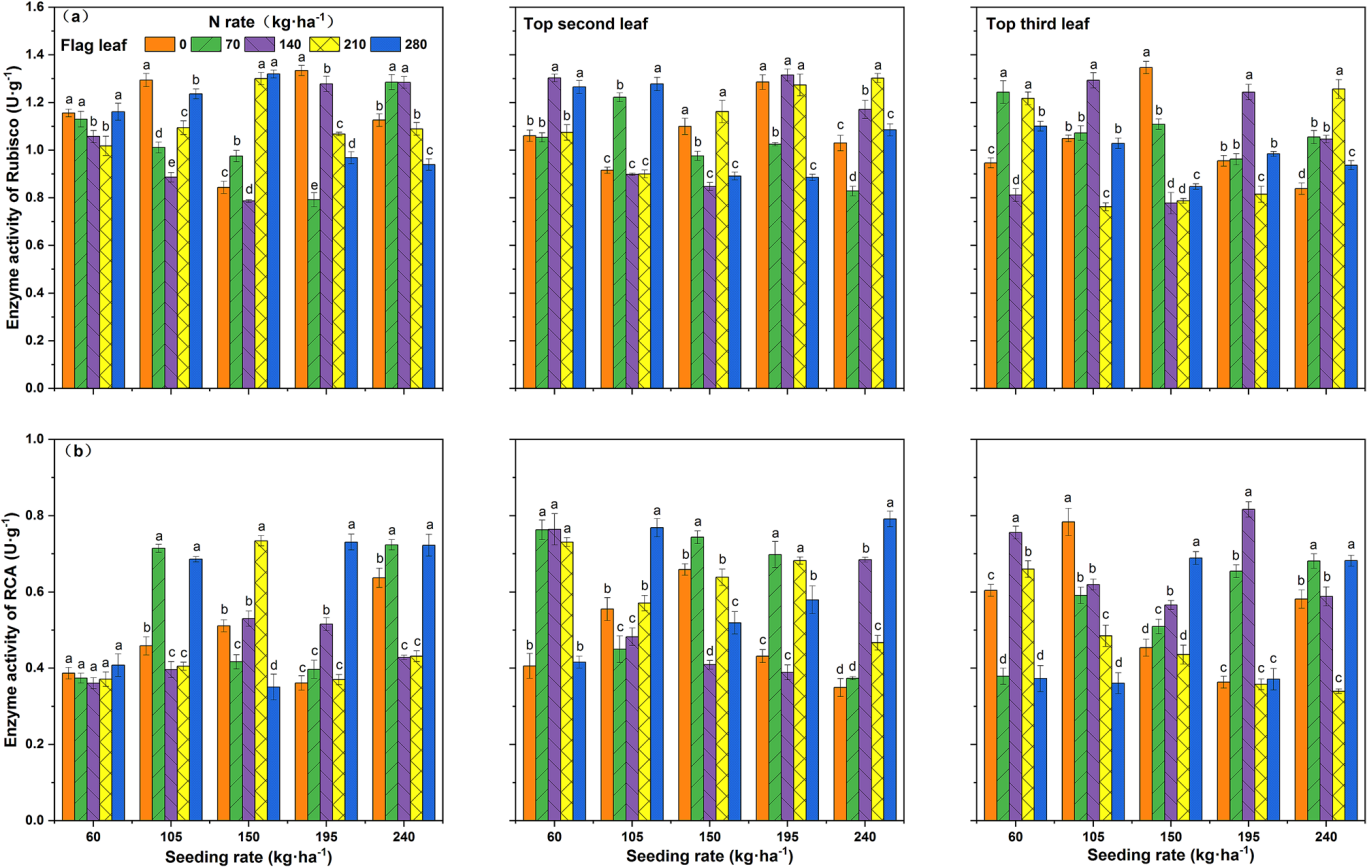
Effects of the interaction between seeding rate and nitrogen rate on Rubisco and RCA activities at different leaf positions of the rice population 10 days after flowering under dry cultivation. (**a**) Rubisco activity in the flag leaf, first second leaf, and first third leaf. (**b**) RCA activity of the flag leaf, first second leaf, and first third leaf. Different letters with the same sowing amount indicate a significant difference at P < 0.05. The figures were prepared and combined using Origin 2018 (www.originlab.com).

The average level of Rubisco activity was higher in the flag leaf and the inverted second leaf at all seeding rates. The RCA activity among leaf positions at A1–A4 seeding rates was higher in the inverted second leaf and the inverted third leaf and lower in the flag leaf. The activities of Rubisco and RCA were higher in the inverted second leaf at the A4 seeding rate. Rubisco activity of the flag leaf under A1–A3 seeding rates was higher under B1 and B5 nitrogen rates. Under A4–A5 seeding rates, Rubisco activity tended to decrease by continuing to increase the nitrogen rate after reaching B3–B4. Higher RCA activity could be achieved at the three-leaf position under B2 or B3 nitrogen rates. The overall Rubisco activity at the three-leaf position was higher in the A4B3 treatment with respect to the interaction between seeding rate and nitrogen rate. The overall RCA activity at the three-leaf position was higher in A4B2 and A4B3 treatments at the A4 seeding rate (Fig. 3).

### *rbcL* and* rca* gene expressions

**Figure 4:**
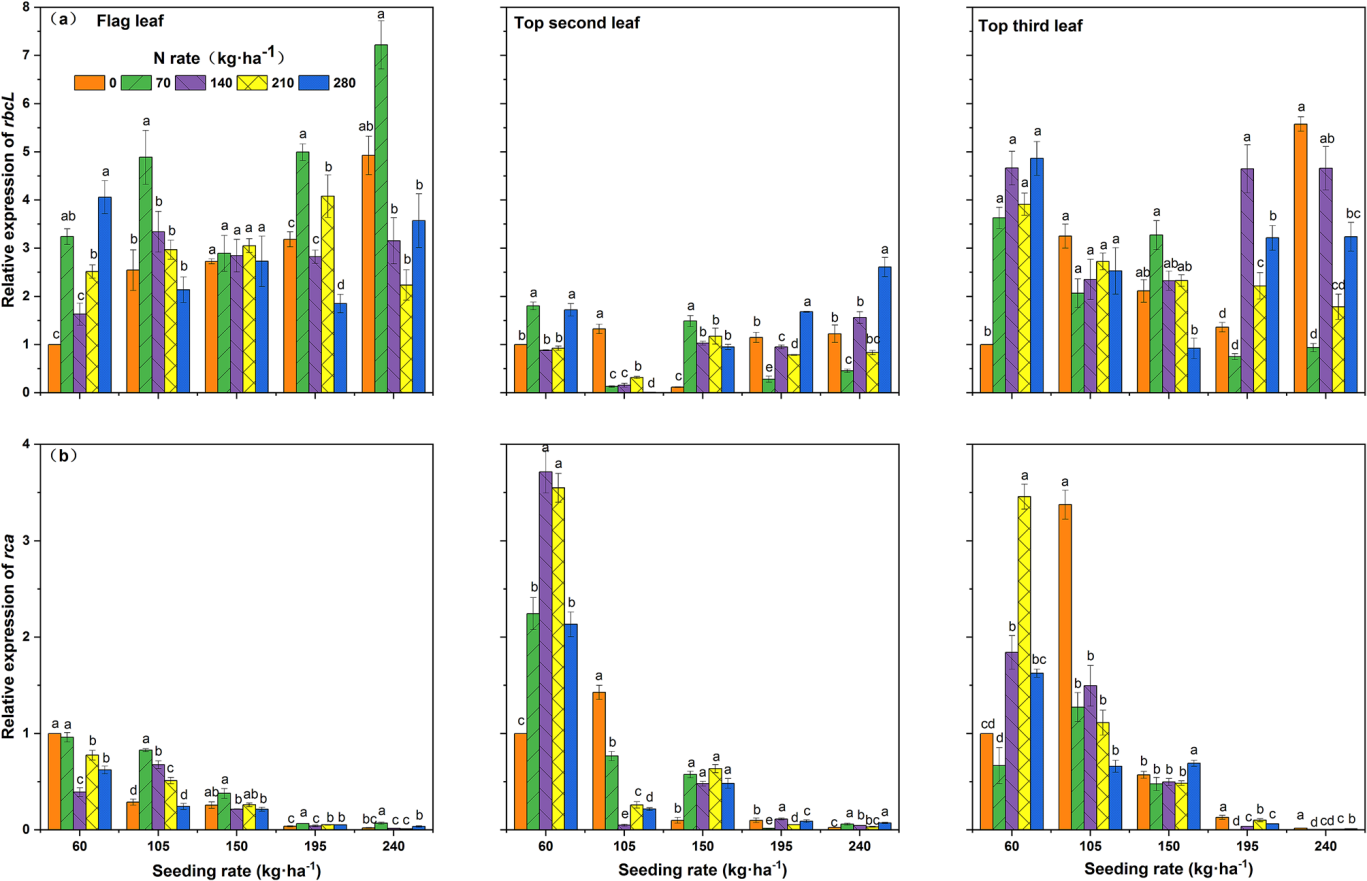
Effects of interaction between seeding rate and nitrogen rate on *rbcL* and *rca* gene expressions at different leaf positions of the rice population 10 days after flowering under dry cultivation. (**a**) The expression level of the *rbcL* gene in the flag leaf, the top two leaves, and the top three leaves. (**b**) The expression level of the *rca* gene in the flag leaf, the top two leaves, and the top three leaves. Different letters with the same sowing amount indicate a significant difference at P < 0.05. The figures were prepared and combined using Origin 2018 (www.originlab.com).

With increasing seeding rate, the total *rbcL* expression in the upper three leaves followed a decreasing and then an increasing trend, while the *rca* expression followed a decreasing trend. The mean level of *rbcL* expression at each seeding rate was elevated in the inverted third leaf and the flag leaf. The mean level of *rca* expression under A1–A4 seeding rates had higher levels in the inverted second leaf and the inverted third leaf and lower levels in the flag leaf among leaf positions. With regard to the interaction between seeding rate and nitrogen rate, the total *rbcL* expression at the three-leaf position was highest at B5 at A1 seeding rate and *rca* expression was highest at B4. The total expressions of *rbcL* and *rca* at the three-leaf position were higher in B1 and B2 at the seeding rates A2, A3, and A5. The highest *rbcL* expression was found in A4B3 treatment under A4 seeding rate (Fig. 4).

### Nitrogen accumulation and utilization

**Figure 5:**
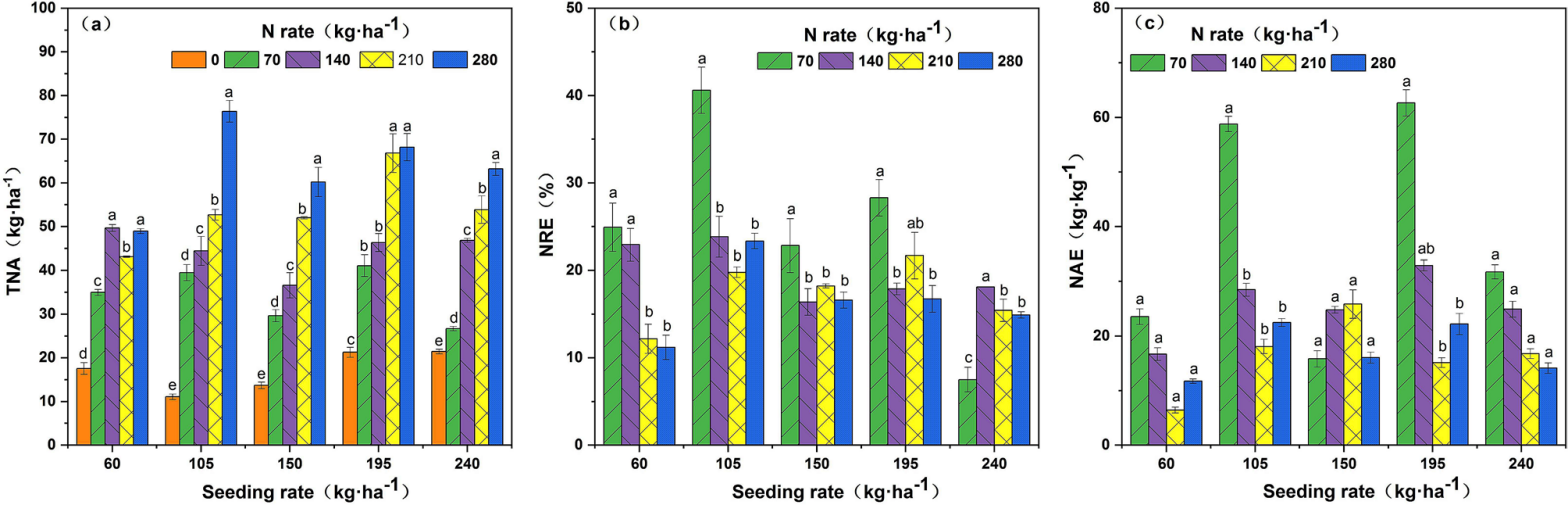
Effects of the interaction between seeding rate and nitrogen rate on nitrogen accumulation and nitrogen use efficiency of rice under dry cultivation. *TNA* total N accumulation, *NRE* N recovery efficiency, *NAE* N agronomic efficiency. Different letters with the same sowing amount indicate significant differences at P < 0.05. The figures were prepared and combined using Origin 2018 (www.originlab.com).

The contradiction between high nitrogen accumulation and low utilization can be reconciled by understanding the interaction between seeding rate and nitrogen rate. The average levels of TNA(total N accumulation), NRE, and NAE(N agronomic efficiency) in A1–A2 range followed an increasing trend with increasing seeding rate, while the average levels of A4–A5 followed a decreasing trend, which were higher at A2 and A4 and lower at A1, A3, and A5 for different seeding rates. No significant difference was found in TNA at B3 and B5 for A1 seeding rate, but the highest value of TNA was found in B4–B5 under A2–A5 seeding rates. NRE was higher in the B2–B3 range under A1 and A2 seeding rates. NRE decreased with increasing nitrogen rate in the B4–B5 range at A3–A5 seeding rates. The NAE under A1 and A2 conditions was highest at B2 and lower at B4 and B5. NAE was lower under A4 and A5 seeding rates than under B4 and B5 levels (Fig. 5).

## Discussion

Panicle number, spikelets per panicle, filled grain rate, and 1000-grain weight are the four components of yield. Increasing the panicle number and the number of grains while retaining a stable filled grain rate and 1000-grain weight, is assumed as the key to increase rice yield^29–31^. Nitrogen supplementation and density control are the two most important crop management practices that significantly affect rice growth and yield formation^32–34^. In this study, the effective panicle number and spikelets per panicle were positively correlated with yield (r = 0.750**; r = 0.632**), and the interaction of seeding rate and nitrogen rate had a significant effect on panicle number and spikelets per panicle (*P* = 0.018; *P* < 0.01). The number of effective panicles could be increased by increasing the nitrogen rate, while the number of plants could be increased by increasing their density. Increases of panicle number and spikelets per panicle were reported to restrict each other^8^. In this study, with increasing seeding rate, the panicle number increased and spikelets per panicle decreased. The nitrogen rate needed to achieve the highest yield under each seeding rate differed, and the interaction between the seeding rate and the nitrogen rate can coordinate the comprehensive advantages of panicle number and spikelets per panicle, and increase group storage capacity to achieve higher yield.

With regard to the external characteristics of photosynthetic organs of dominant populations, it has been shown that a reasonable sowing density can regulate the population structure of rice and alleviate the contradiction between individual development and population growth^35^. Increased nitrogen fertilization increases the photosynthetic potential after tasseling^36^. In the present study, the population photosynthetic potential was found to be highly significantly and positively correlated with yield 10 days after flowering (r = 0.854**). Increasing the amount of seeding and nitrogen application increased the population photosynthetic potential and enhanced the population photosynthetic potential. Excessive N application can lead to excessive population density and plant collapse^37–39^, and can also reduce the N fertilizer utilization, which decreases ecological benefits^40–42^. In this study, severe collapse occurred in groups with excessive plant height at seeding rates of 195–240 kg·ha^−1^ and at a nitrogen rate exceeding 140 kg·ha^−1^. The nitrogen agronomic utilization efficiency was highest under a combination of a seeding rate of 195 kg·ha^−1^ and a nitrogen rate of 140 kg·ha^−1^. In this experiment, the excessive increase of photosynthetic potential and plant height caused by excessive seeding rate and nitrogen rate may have been the main cause of population lodging. Excessive photosynthetic potential increased the competition among individuals and decreased the ventilation and light transmission ability of the population. Excessive nitrogen rate increased plant height and decreased the ability of the population to resist lodging. It has been shown that the plant shape of rice directly seeded at the flush stage is characterized by high leaf position, longer leaf length of the upper three leaves, smaller leaf-stalk angle, and an external morphology that exhibits elongated leaf shape and compact plant shape^43^. Increasing N application and population density promotes plant growth and increases the photosynthetic potential as well as interception of photosynthetically active radiation, which forms an important prerequisite for the emergence of a dominant populations^44–46^. In this study, a two-way positive correlation was found between the photosynthetic potential of the population 10 days after flowering, as well as between the light energy interception rate of the inverted second leaf and yield. This indicates that the inverted second leaf played an important role in coordinating the light energy interception capacity of the population. It is not reasonable to excessively increase the seeding rate and nitrogen rate in conjunction with a comprehensive analysis of population photosynthetic organ morphology, plant height, collapse, and ecological benefits. A seeding rate of 195 kg·ha^−1^ and a nitrogen rate of 140 kg·ha^−1^ can coordinate the interception of light energy by the inverted second leaf. Thus, this improves the interception of light energy by the population, coordinates the photosynthetic potential of the population, while at the same time, achieves the appropriate plant level to obtain a dominant population without collapse, but with high yield and high ecological efficiency.

In terms of the photosynthetic physiological characteristics of the dominant group, Pn level and Chl content are important indicators for evaluating the photosynthetic capacity of crops^47,48^. It has been shown that in rice, the highest chlorophyll content, Rubisco enzyme activity, and rca gene expression in sword leaves were reached 5–15 days after flowering^49^. In the present experiment, two highly significant positive correlations were found for Pn and Chl among leaf positions, with higher Pn and Chl in the flag leaf and the inverted second leaf. PN and Chl of the inverted second leaf had the highest correlation with yield (r = 0.488**; r = 0.679**). RCA activity can regulate the initial Rubisco activity, and Rubisco activity is highly positively correlated with Pn. Rubisco activity decreases faster than photosynthesis and chlorophyll content during leaf senescence^50,51^. In the present study, the inverted second leaf RCA enzyme activity was highly positively correlated with Rubisco activity, and the Rubisco activity of the inverted second leaf was positively correlated with Pn. The flag leaf Pn and Chl were higher but not strongly synchronized with photosynthetic enzyme activity, indicating that, in addition to having higher Pn and Chl, the synergism between Pn and Rubisco is particularly important in photosynthetic physiological characteristics. As a key photosynthetic enzyme, the activity of Rubisco is affected by the external environment^52,53^. In this study, the activity of Rubisco was positively correlated with photosynthetic potential, and the positive correlation between light energy interception rate and Rubisco activity was highest in different leaf positions in the inverted second leaf. This indicates that the photosynthetic enzyme activity, net photosynthetic rate, and chlorophyll content of rice under dry cultivation in the inverted second leaf 10 days after flowering were synchronized and coordinated. This, in turn, enhanced the yield potential of the population.

It has been found that once plants are exposed to light, the transcript levels of *rbcL* and *rca* increase rapidly, and Rubisco activity increases similarly^54^. Suitable supplementation with nitrogen fertilizer can meet the requirements of Rubisco synthesis, but sometimes, the transcription and translation of the Rubisco gene are not synchronized^49^. In this study, the gene expression of the inverted third leaf of the dominant population was relatively high, which may have been caused by the decreased gene expression in the sword leaf and the inverted second leaf. In this study, the changes in photosynthetic index values and the synergisms among the indexes were related to the leaf position. The expression of the *rbcL* gene in the inverted third leaf was positively correlated with the Rubisco enzyme activity in the inverted second leaf. Positive correlation was found between *rca* gene expression and RCA activity in the inverted second and inverted third leaf. The *rca* gene expression and RCA activity were lower and the synergism of gene, enzyme activity, and net photosynthetic rate changes were lower in the flag leaf. The flag leaf of rice showed gradual senesce during tassel setting, which was initially characterized by a decrease in photosynthetic rate and protein content, followed by a decrease in chlorophyll and RNA^49^. In this study, 10 days after flowering, the photosynthetic physiological characteristics of three leaves indicated high gene expression level in lower leaves, higher enzyme activity in middle leaves, and stable net photosynthetic rate and chlorophyll content in upper leaves. The change of photosynthetic index could be ordered from gene to enzyme activity, then to net photosynthetic rate and chlorophyll, from the flag leaf to the inverted second leaf and then to the inverted third leaf.

**Figure 6:**
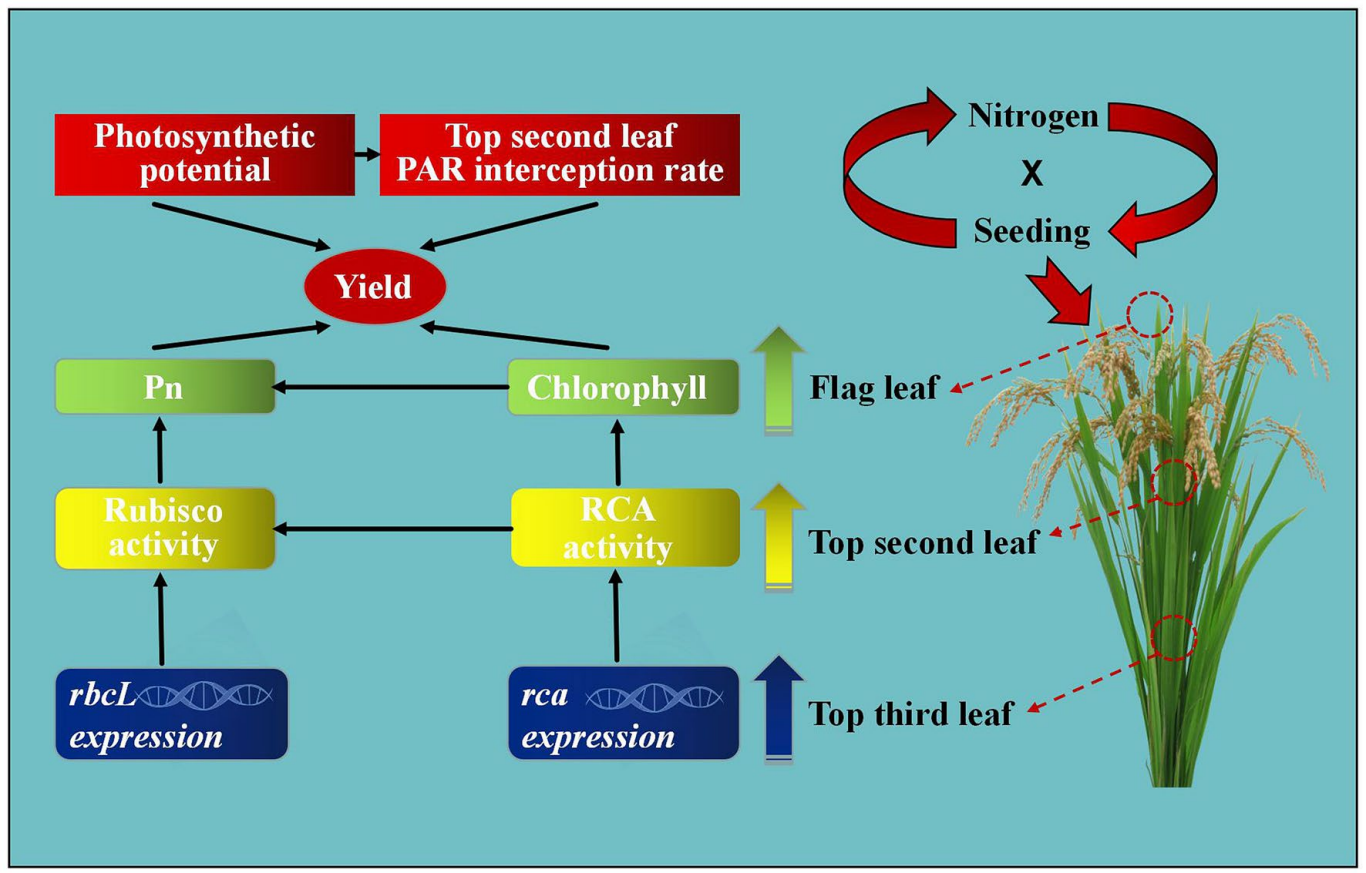
Mechanism underlying the formation of dominant rice populations under dry cultivation. The figures were prepared and combined using Origin 2018 (www.originlab.com).

Through investigating the interaction of seeding rate and nitrogen rate, this study obtained a theoretical and practical basis for constructing a dominant rice population under dry cultivation. Breeding objectives and an optimization system was formulated for rice under dry cultivation, and a theoretical basis for the coordination of the synchronization of genes, enzyme activities, and photosynthetic indexes of the upper three leaves of rice under dry cultivation was provided by means of genetic engineering and proteomics (Fig. 6). This study used strip sowing. To identify the differences of seeding rate and nitrogen rate between strip and hole sowing require further investigation.

## Conclusions

Threshold values were identified for photosynthetic potential and plant height 10 days after flowering in rice under dry cultivation. Exceeding these thresholds will lead to uncoordinated distribution of light energy in the upper three leaves of the population, large population competition, greed, and late maturity, all of which lead to a decline in yield and the collapse of the population. The inverted second leaves show superior in light energy interception and have a greater impact on population photosynthetic coordination. Changes in photosynthetic physiological indicators could be ordered from gene to enzyme activity to net photosynthetic rate and chlorophyll, and the spatial variation is from the upper leaf to the lower leaf. An important tool to construct a dominant rice population under dry cultivation is the protection of the flag, the promotion of the second flag, and the stabilization of the third flag. This maintains the photosynthetic capacity of the flag leaf, promotes the photosynthetic enzyme activity in the inverted second leaf, and stabilizes the photosynthetic gene expression in the inverted third leaf. This study showed that under the interaction of 195 kg·ha^−1^ seeding rate and 140·ha^−1^ nitrogen rate, a dominant population could be built with coordinated light energy distribution in the upper three leaves, moderate plant size, large yield advantage, and high nitrogen agronomic utilization under reciprocal cropping. These values can be used as a references for rice cultivation under dry conditions for central Jilin Province.

## Materials and methods

### Test site and test materials

The trial was conducted in 2019 and 2020 at the National Crop Variety Validation Characterization Station on the campus of Jilin Agricultural University, Changchun, Jilin Province, China (125° 39 E, 44° 46 N). The organic matter content of soil was 18.7%, alkaline dissolved nitrogen was 117.02 mg·kg^−1^, fast-acting phosphorus was 41.11 mg·kg^−1^, fast-acting potassium was 245.16 mg·kg^−1^, and the pH was 6.2. The cumulative temperature and rainfall for the whole reproductive period were 2851.9 °C and 664.4 mm in 2019 and 2920.2 °C and 593.1 mm in 2020.

The test variety was Suijing 18 (China Rice Data Center, No. 2014021, https://www.ricedata.cn/variety/varis/614593.htm), provided by researcher Shoujun Nie of Suihua Branch, Heilongjiang Provincial Academy of Agricultural Sciences. The growing period is 134–135 days, and it is one of the major varieties in the region. A preliminary experiment verified that Suijing18 is a high-quality variety with strong drought resistance suitable dry cultivation in central Jilin Province.

### Experimental design

The experiment used a two-factor split-zone design, with five levels of seed sowing at 60 kg·ha^−1^ (A1), 105 kg·ha^−1^ (A2), 150 kg·ha^−1^ (A3), 195 kg·ha^−1^ (A4), and 240 kg·ha^−1^ (A5) in the main zone. Five levels of nitrogen application at 0 kg·ha^−1^ (B1), 70 kg·ha^−1^ (B2), 140 kg·ha^−1^ (B3), 210 kg·ha^−1^ (B4), and 280 kg·ha^−1^ (B5) were applied in the secondary zone, respectively. The plot area was 20 m^2^ with three field replications; the seeds were manually mechanical strip sown. Seeds were sown on May 6 in both years, coated before sowing, and dried in the shade until they stopped to be sticky. The sowing row spacing was 25 cm for all treatments. All fertilizers were applied at once as base fertilizer. 75 kg·ha^−1^ each of phosphate (in P_2_O_5_) and potash (in K_2_O) were used as basal fertilizers for each treatment and urea was used as basal fertilizer for nitrogen. Ridges were built around each treatment plot to prevent water and fertilizer loss. During the whole reproductive period, the experiment mainly relied on natural rainfall, and only used sprinklers for uniform water replenishment during drought and periods of critical water demand. Other field management measures were conducted according to the general high-yielding field model to ensure consistent management across all experimental plots.

### Yield and yield components

Three rows of 4 m each were selected as survey points in each plot before harvest. The average was used to calculate the effective number of panicles. Fifteen representative plants were taken for seed testing, and the spikelets per panicle, filled grain rate, and 1000-grain weight were measured.

### Net photosynthetic rate

The Pn was measured 10 days after flowering on the main stem rapier leaves, the inverted second leaves, and the inverted third leaves. A Li-6400XT photosynthesizer(Manufactured by Li-Cor Corporation, USA.) was used with a built-in fixed light source and a light quantum density setting of 1200 µmol·m^−2^·S^−1^. The rates were measured between 9:00 and 11:30 a.m. on a clear and windless day, with three replicates per treatment. The mean values were calculated.

### Chlorophyll

10 days after flowering, three fully expanded leaves of the main stem, and the inverted second and third leaves were harvested. 0.1 g of fresh samples of cut and mixed leaves were extracted with 95% ethanol, and chlorophyll a and chlorophyll b contents (mg/g) were calculated after measuring the absorbance at 665 nm and 649 nm using a spectrophotometer (Tu-1810 is manufactured by Beijing Purkinje General Instrument Co.,Ltd.).

### Rubisco and RCA activity

The fresh upper three leaves were harvested on day 10 after flowering and were used for the determination of Rubisco and RCA enzyme activities. Each treatment was replicated three times and tested by Qingdao Sci-Tech Quality Testing Co. The company is located in Shandong Province, China.

Rubisco enzyme activity assay: A solid phase antibody was built by coating a microtiter plate with purified plant Rubisco 1,5-diphosphate antibody. Plant Rubisco was added sequentially to the microtiter wells coated with the monoclonal antibody, and was then combined with HRP-labeled Rubisco antibody to form an antibody-antigen-enzyme-labeled antibody complex. This complex was thoroughly washed and then colored with the substrate TMB. TMB was converted to blue by HRP enzyme and to the final yellow color by the action of an acid. The shade of color was positively correlated with the Rubisco content in the sample. The absorbance (i.e., OD value) was measured at 450 nm using an enzyme standardizer, and the concentration of Rubisco activity in the samples was calculated via the standard curve.

RCA enzyme activity assay: Purified plant Rubisco activase (RCA) antibody was used to coat the microtiter plate to obtain a solid phase antibody. Plant RCA was added sequentially to the microtiter wells coated with the monoclonal antibody, and then combined with HRP-labeled RCA antibody to form an antibody-antigen-enzyme-labeled antibody complex. This complex was also thoroughly washed and then colored with the substrate TMB. The shade of color was positively correlated with the plant RCA in the sample. The absorbance was measured at 450 nm using an enzyme marker and the concentration of phyto-RCA activity in the samples was calculated from the standard curve.

### *rbcL* and *rca* gene expressions

Fresh upper three leaves, 10 days after flowering, were harvested for RNA extraction. A small amount of these leaves was cut and ground in a pre-cooled mortar, and about 0.1 g of powder was weighed for RNA extraction. RNA extraction of the leaf material was performed using the Trizol method, followed by reverse transcription using a reverse transcription kit (Supplied by TAKARA in Japan). The procedure was performed according to the instructions of the TAKARA Trizol and PrimeScriptTMRT reagent kit with gDNA Eraser. The cDNA was diluted 1:3 before its use for qPCR.

The primer sequences of the target genes rbcL and rca as well as the internal reference gene actin used in this study were obtained from the report of Hongling Tang^49^. The primer information is shown in Table 2.

**Table 2:** Sequences of primers for real-time quantitative PCR.

Gene name	Gene ID	Forward primer(5*′*–3*′*)	Reverse primer(5*′*–3*′*)
Actin	X16280	GAGACCTTCAACACCCCTGCTA	ATCACCAGAGTCCAACACATTACCT
rbcL	D00207	CTTGAATGCGACTGCAGGTA	GAAGAAGTAGGCCGTTGTCG
rca	U74321	GACTGGTTCCTTCTACGGTTC	TGCTTGCTGTGCTCCTTG

Fluorescent quantitative PCR was measured using an ABI stepone plus real-time fluorescent quantitative PCR instrument with the TAKARA SYBRPremix Ex Taq II kit. The following steps were applied: All qPCR assays were performed with an ABI 7300 PCR system (Applied Biosystems) in a 20 µL reaction volume. This reaction volume containing 1 µl of template (10 ng/lg DNA), SYBR green PCR mastermix, and each primer. The following hot procedures were applied: A single DNA polymerase activation cycle at 95 °C for 10 min, followed by 40 amplification cycles at 95 °C for 30 s (denaturation step) and 60 °C for 1 min (annealing-extension step). The dissolution curve procedure was: 95 °C for 15 s, 60 °C for 1 min, and 95 °C for 15 s. The technique was repeated three times. The standard curve was obtained by diluting the sample cDNA in a concentration gradient. The reference gene was used as the standard for relative quantification by the 2^−△△Ct^ method when the amplification efficiencies of the target gene and the reference gene were similar.

### Nitrogen accumulation and nitrogen use efficiency

Samples were taken at both full heading stage and mature stage. Three plants were selected from each plot and were decomposed into to stems, leaves, and panicles. Then, samples were sterilized at 105 °C for 30 min, and dried to constant weight at 80 °C. After grinding, the samples were digested with H_2_SO_4_–H_2_O_2_, and the nitrogen content of each organ sample was determined by an automatic Kjeldahl nitrogen analyzer (Kjeltec 8100 manufactured by FOSS USA).

### Data calculation and statistical analysis

Since the data were basically the same for both years, the analysis was performed with 2020 data.

1. Nitrogen recovery efficiency (%) = (nitrogen accumulation of plants in the nitrogen application zone − nitrogen accumulation of plants in the nitrogen free zone)/nitrogen application × 100.
2. Nitrogen agronomic efficiency (kg/kg) = (seed yield in the nitrogen application area − seed yield in nitrogen free area)/nitrogen application.
3. Photosynthetic potential (m^2^·day/m^2^) = (L_1_ + L_2_) × (t_2_ − t_1_)/2, where L1 and L2 represent the leaf areas measured before and after exposure to light, and t1 and t2 are the times of the two measurements before and after.
4. Light energy interception rate (%) of sword leaves = (light intensity at sword leaves − light intensity at inverted second leaves)/light intensity at sword leaves × 100.
5. Light energy interception rate of inverted second leaves = (light intensity at inverted second leaves − light intensity at inverted third leaves)/light intensity at inverted second leaves × 100.

Microsoft Excel 2017 was used for data entry and organization, SPSS 21.0 software was used for data analysis, and Origin 2018 was used for plotting.

## Data Availability

All data generated or analyzed during this study are included in this published article (and its Supplementary Information file).
